# Prevalence of hyperuricemia and its related risk factors among preschool children from China

**DOI:** 10.1038/s41598-017-10120-8

**Published:** 2017-08-25

**Authors:** Nan Li, Shuang Zhang, Weiqin Li, Leishen Wang, Huikun Liu, Wei Li, Tao Zhang, Gongshu Liu, Yuexin Du, Junhong Leng

**Affiliations:** Tianjin Women and Children’s Health Center, Tianjin, China

## Abstract

To estimate the prevalence of hyperuricemia and its major risk factors among Chinese preschool children. A cross-sectional survey was carried out in six central urban districts of Tianjin from March to June 2015. A cluster sampling was employed to obtain a random sample of preschool children. A total of 4073 children aged from 3 to 6 years participated in this survey. Logistic regression was used to obtain odds ratios and 95% confidence intervals. The overall mean serum uric acid concentrations was 243.0 ± 53.2 μmol/L, corresponding to a mean concentrations of 247.3 ± 53.7 μmol/L among boys and 238.3 ± 52.4 μmol/L among girls. The overall prevalence of serum uric acid ≥ 310 μmol/L among children was 10.1%. Boys, obesity, diastolic blood pressure, and serum triglyceride concentrations were associated with the increased risk of hyperuricemia in univariable models, although the statistically significant association between diastolic blood pressure, serum triglyceride concentrations and hyperuricemia disappeared in multivariable models. The prevalence of hyperuricemia among children aged from 3 to 6 years was relatively high. Several metabolic syndrome components were associated with the risk of hyperuricemia.

## Introduction

Serum uric acid and the prevalence of hyperuricemia in adults have been rising over the past decades^1–3^. The overall prevalence of hyperuricemia among United States (US) adults has increased from 18.2% in 1988–1994 to 21.4% in 2007–2008^3^. A few studies showed that hyperuricemia in adults was associated with metabolic syndrome and its components, including type 2diabetes and coronary artery disease^3–8^. The US National Health and Nutrition Examination Survey (NHANES) of 1988–1994 found that the prevalence of metabolic syndrome increased substantially with increasing serum uric acid levels^3^. Meanwhile, some studies showed that the indices of blood pressure (BP), triglyceride, and obesity were important risk factors of serum uric acid^7, 8^.

Some studies also reported the association between the elevated serum uric acid level and its risk for metabolic syndrome and its components in school age children population. Recently, a cross-sectional prevalence study indicated that hyperuricemia among 148 obese or overweight adolescents was strongly associated with metabolic syndrome^9^. Another cross-sectional analysis of 1370 US children and adolescents aged 12 to 17 years also found that serum uric acid concentrations were strongly associated with the prevalence of the metabolic syndrome and several of its components^10^. However, to date, studies on the prevalence of hyperuricemia in preschool children are lacking and the relationship between hyperuricemia in preschool children and the components of metabolic syndrome is unknown. The aim of the present study was to estimate the prevalence of hyperuricemia and its related risk indicators among the preschool children in urban districts of Tianjin, China.

## Results

We recruited 4073 children between 3 and 6 years old with an average age of 5.6 ± 0.8 years old and an average BMI of 15.9 ± 2.2 kg/m^2^, 9.4% were overweight and 12.9% were obese.

The overall mean serum uric acid concentration was 243.0 ± 53.2 μmol/L. The averages of the data studied were: systolic blood pressure: 97.8 ± 10.9 mmHg, diastolic blood pressure: 59.5 ± 8.4 mmHg, fasting glucose: 4.87 ± 0.37 mmol/L, total cholesterol 4.28 ± 0.70 mmol/L, triglycerides: 0.75 ± 0.33 mmol/L. 2.5% had an impaired fasting glucose, and 9.6% and 1.4% were diagnosed with high total cholesterol and high triglycerides, respectively (Table 1). There were no significantly differences on education levels (mother or father) and family income between children with normal serum uric acid (<310 μmol/L) and higher serum uric acid (≥310 μmol/L). The history of gout of child’s mother and father were about 0.05% (2/4059) and 1% (40/4073), respectively, and there was also no significantly difference between children with normal serum uric acid (<310 μmol/L) and higher serum uric acid (≥310 μmol/L). The distributions of uric acid levels by age, sex and BMI are summarized in Table 2. The mean level of uric acid in boys (247.3 ± 53.7 μmol/L) was significantly higher than that in girls (238.3 ± 52.4 μmol/L), especially among those children aged more than 5 years old. Compared with children with normal weight, children with overweight or obesity had a higher concentrate of serum uric acid in both boys and girls.

**Table 1:** Clinical and anthropometric characteristics.

	M ± SD
Age (years)	5.6 ± 0.8
BMI (kg/m^2^)	15.9 ± 2.2
SBP(mmHg)	97.8 ± 10.9
DBP(mmHg)	59.5 ± 8.4
UA (μmol/L)	243.0 ± 53.2
FG (mmol/L)	4.87 ± 0.37
TC (mmol/L)	4.28 ± 0.70
TG (mmol/L)	0.75 ± 0.33
Overweight* (%)	9.4
Obesity* (%)	12.9
**Hypertension** ^#^	
High SBP (%)	15.7
High DBP (%)	7.0
FG ≥ 5.6 mmol/L (%)	2.5
TC ≥ 5.17 mmol/L (%)	9.6
TG ≥ 1.70 mmol/L (%)	1.4

*Overweight was defined as weight-for-BMI ≥ 85th percentile (≥1.035 Z score); Obesity was defined as weight-for-BMI ≥ 95th percentile (≥1.645 Z score); Sex-specific weight-for-BMI percentiles were based on WHO growth reference (World Health Organization, 2006).

^#^Hypertension was defined in accordance with the blood pressure reference standards for Chinese children as systolic or diastolic BP ≥ 95th percentile for age and sex.

Abbreviations: BMI body mass index, UA uric acid, SBP systolic blood pressure, DBP diastolic blood pressure, FG fasting glucose, TC total cholesterol, TG triglyceride.

**Table 2:** Distribution of uric acid by age, sex, and BMI.

	Boy		Girl		Total		*P for difference*
	N	M ± SD	N	M ± SD	N	M ± SD	
Total	2140	247.3 ± 53.7	1933	238.3 ± 52.4	4073	243.0 ± 53.2	<0.001
**Age (years)**							
6− < 7	783	248.1 ± 53.6	671	237.6 ± 48.6	1454	243.3 ± 51.6	<0.001
5− < 6	921	248.6 ± 53.9	819	239.9 ± 52.1	1740	244.5 ± 53.2	0.001
4− < 5	289	242.9 ± 55.9	292	238.8 ± 61.3	581	240.8 ± 58.7	0.409
3− < 4	147	243.1 ± 48.2	151	231.9 ± 51.3	298	237.4 ± 50.0	0.053
*P for trend*		0.310		0.365		0.130	
**Overweight/Obesity**							
Normal	1580	242.3 ± 51.5	1585	235.2 ± 52.5	3165	238.7 ± 52.1	<0.001
Overweight*	206	251.8 ± 52.7	177	246.1 ± 48.9	383	249.1 ± 51.0	0.275
Obesity*	354	267.0 ± 59.0	171	259.6 ± 49.0	525	264.6 ± 56.0	0.155
*P for trend*		<0.001		<0.001		<0.001	

Data are means (SD).

*Overweight was defined as weight-for-BMI ≥ 85th percentile (≥1.035 Z score); Obesity was defined as weight-for-BMI ≥ 95th percentile (≥1.645 Z score); Sex-specific weight-for-BMI percentiles were based on WHO growth reference (World Health Organization, 2006).

The prevalence of hyperuricemia among children in different subgroups is shown in Table 3. The overall prevalence of hyperuricemia among preschool children was 10.1%. The prevalence of hyperuricemia among boys (11.8%) was significantly higher than that among girls (8.3%) (*P* < 0.001). Compared with the prevalence of hyperuricemia among children with normal weight (8.7%), the prevalence increased among children with overweight (9.9%) and obesity (18.9%) (*P* for trend <0.001). Children with higher diastolic BP or higher serum triglyceride concentrations (≥1.70 mmol/L) had higher prevalence of hyperuricemia (*P* < 0.05).

**Table 3:** Prevalence of hyperuricemia among 4073 children by different subgroups.

	Hyperuricemia ≥ 310 μmol/L		
	N	%	*P*
Total	413	10.1	
Sex			<0.001
Girl	161	8.3	
Boy	252	11.8	
Age (years)			0.113
6− < 7	137	9.4	
5− < 6	198	11.4	
4− < 5	55	9.5	
3− < 4	23	7.7	
Overweight/Obesity			<0.001
Normal	276	8.7	
Overweight*	38	9.9	
Obesity*	99	18.9	
SBP			0.078
Normal	262	9.6	
Hypertension^#^	60	11.8	
DBP			0.024
Normal	290	9.7	
Hypertension^#^	32	14.2	
FG			0.338
FG < 5.6 mmol/L	401	10.1	
FG ≥ 5.6 mmol/L	12	11.8	
TC			0.315
TC < 5.17 mmol/L	370	10.1	
TC ≥ 5.17 mmol/L	43	10.9	
TG			0.004
TG < 1.70 mmol/L	400	10.0	
TG ≥ 1.70 mmol/L	13	22.8	

*Overweight was defined as weight-for-BMI ≥ 85th percentile (≥1.035 Z score); Obesity was defined as weight-for-BMI ≥ 95th percentile (≥1.645 Z score); Sex-specific weight-for-BMI percentiles were based on WHO growth reference (World Health Organization, 2006).

^#^Hypertension was defined in accordance with the blood pressure reference standards for Chinese children as systolic or diastolic BP ≥ 95th percentile for age and sex.

Abbreviations: SBP systolic blood pressure, DBP diastolic blood pressure, FG fasting glucose, TC total cholesterol, TG triglyceride.

Table 4 shows the association between the potential risk factors and hyperuricemia risk. Boys (OR: 1.47, 95% CI: 1.19–1.81), obesity (OR: 2.43, 95% CI: 1.89–3.13), higher diastolic BP (OR: 1.54, 95% CI: 1.04–2.28), and higher serum triglyceride concentrations (OR: 2.67, 95% CI: 1.43–5.00) were positively associated with the risk of hyperuricemia risk in univariable analysis model (model 1). After adjustment for all potential influencing factors, boys and obesity remained significantly associated with the risk of hyperuricemia risk (model 2).

**Table 4:** Odd ratios (95% confidence intervals) of hyperuricemia by different factors.

	Hyperuricemia ≥ 310 μmol/L			
	Model 1		Model 2	
	OR (95 CI)	*P*	OR (95 CI)	*P*
Sex				
Girl	1.00		1.00	
Boy	1.47 (1.19–1.81)	<0.001	1.33 (1.03–1.73)	0.030
Age (years)				
6− < 7	1.00		1.00	
5− < 6	1.23 (0.98–1.55)	0.073	1.25 (0.96–1.63)	0.093
4− < 5	1.01 (0.72–1.40)	0.975	0.55 (0.27–1.11)	0.095
3− < 4	0.80 (0.51–1.27)	0.353	1.33 (0.58–3.04)	0.502
Overweight/ Obesity				
Normal	1.00		1.00	
Overweight*	1.15 (0.81–1.65)	0.435	1.35 (0.89–2.05)	1.353
Obesity*	2.43 (1.89–3.13)	<0.001	2.55 (1.87–3.48)	<0.001
SBP				
Normal	1.00		1.00	
Hypertension^#^	1.26 (0.93–1.70)	0.131	0.84 (0.58–1.22)	0.839
DBP				
Normal	1.00		1.00	
Hypertension^#^	1.54 (1.04–2.28)	0.031	1.42 (0.89–2.27)	0.139
FG				
FG < 5.6 mmol/L	1.00		1.00	
FG ≥ 5.6 mmol/L	1.19 (0.64–2.19)	0.583	0.88 (0.39–1.97)	0.757
TC				
TC < 5.17 mmol/L	1.00		1.00	
TC ≥ 5.17 mmol/L	1.10 (0.79–1.54)	0.580	1.07 (0.68–1.70)	0.773
TG				
TG < 1.70 mmol/L	1.00		1.00	
TG ≥ 1.70 mmol/L	2.67 (1.43–5.00)	0.002	1.76 (0.81–3.83)	0.153

Model 1 did not adjust for any variables.

Model 2 further adjusted for history of mother and father regarding gout, education of mother and father, family income, and health history of parents in addition to all variables listed in the model.

*Overweight was defined as weight-for-BMI ≥ 85th percentile (≥1.035 Z score); Obesity was defined as weight-for-BMI ≥ 95th percentile (≥1.645 Z score); Sex-specific weight-for-BMI percentiles were based on WHO growth reference (World Health Organization, 2006).

^#^Hypertension was defined in accordance with the blood pressure reference standards for Chinese children as systolic or diastolic BP ≥ 95th percentile for age and sex.

Abbreviations: SBP systolic blood pressure, DBP diastolic blood pressure, FG fasting glucose, TC total cholesterol, TG triglyceride.

## Discussion

In this study in Tianjin, China, the prevalence of hyperuricemia (≥310 μmol/L (5.2 mg/dL)) in preschool children was 10.1%. Our results have shown that BMI and triglycerides were the most significant contributing factors to hyperuricemia prevalence, which was in accordance with the previous study^11^.

Most of previous studies focused on the prevalence of hyperuricemia among school age children. A cross-sectional analysis using data from the Nation Health and Nutrition Examination Survey(NHANES)1999–2002 reported that the mean concentration of uric acid among children and adolescents aged 12 to 17 years was 301.9 μmol/L (5.1 mg/dL), and the percentage of serum uric acid ≥ 327 μmol/L (5.5 mg/dL) was 30.2%^10^. Another study reported that the median serum uric acid level in risperidone-naïve children and adolescents with Autism Spectrum Disorder was 5.35 mg/dL and the prevalence of hyperuricemia (>5.5 mg/dL) was 44.70%, higher than normal children and adolescent^12^. The mean serum uric acid level of children aged less than 7 years in our study was 243.0 μmol/L (4.1 mg/dL), which was a little higher than that of Hispanic children aged 5 years (3.9 ± 0.8 mg/dL), but lower than previous results of the NHANES study and risperidone-naïve children and adolescents with Autism Spectrum Disorder^10, 12, 13^. Civantos Modino, S. *et al*. studied the prevalence of hyperuricemia in children with overweight or obesity and analyzed the relation between metabolic syndrome and hyperuricemia^9^. They proposed uric acid level which most is the likely diagnosis of metabolic syndrome corresponded to 5.4 mg/dl in children^9^. Although our results found that serum uric of preschool children was lower than school age children, it was noteworthy that there were still 7.6% of children in our study who had serum uric acid level higher than ≥ 321 μmol/L (5.4 mg/dL).

There was no universally accepted threshold to define hyperuricemia in children and adolescents. Most of the previous studies have used the criteria of hyperuricemia for children and adolescents with their own cut-off values^9, 10, 14, 15^. Among children with overweight and obesity, uric acid’s level, from which a diagnosis of metabolic syndrome was most likely, corresponded to 5.4 mg/dl (area under the curve 0.66, sensitivity: 64% and specificity 62%)^9^. An early report^14^ found that a serum uric acid value > 5.5 mg/dL strongly supported the diagnosis of primary hypertension in children (sensitivity: 87% and specificity 86%). In a Japanese study^15^, hyperuricemia was defined as exceeding the mean plus one standard deviation based on the reference value of each age group. Namely, the cut-off values were 5.9 mg/dL for 6–8 years (both genders), 6.1 mg/dL for 9–11 years (both genders), 7.0 mg/dL (males), and 6.2 mg/dL (females) for 12–14 years. In our study, we used 90^th^ percentile of serum uric acid value (5.2 mg/dL) in our sample as the cut-off value of hyperuricemia. In the further study, it is necessary to determine and evaluate the optimal cut-off values of hyperuricemia in preschool children in Chinese and other population.

Several studies concluded that hyperuricemia was a risk factor for the development of diseases such as obesity, hypertension, hyperlipidemia, and diabetes^16, 17^. In concordance with the previous report^10^, our study also found a significant difference in the prevalence of hyperuricemia between obesity and normal weight children. A longitudinal data from the Bogalusa Heart Study indicated that obesity in childhood was a predictor of elevated serum uric acid in adulthood^18^. Other studies also showed a positive association between obesity and uric acid^17, 19^, and some of them hypothesized that obesity is the main determinant of high serum uric acid values in the general population^20^. It is considered that insulin resistance caused by accumulation of fat is the underlying mechanism between obesity and hyperuricemia^21^. On the other hand, it has also been observed that hyperuricemia precedes the development of obesity^17^. Sautin *et al*.^22^ speculated uric acid contributed to the development of obesity by giving rise to inflammatory and oxidative changes in adipocytes.

Many studies showed that hyperuricemia appeared as a common component in subjects with metabolic syndrome^5, 8, 10^. It is known that adult patients with hyperuricemia had a higher risk of developing metabolic syndrome and this prevalence increased with elevation of plasma uric acid levels^5^. In the present study, elevated serum uric acid level was associated with a higher risk of higher diastolic BP and higher serum triglyceride concentrations. Several large studies reported an elevated serum uric acid could predict the development of hypertension in children^14, 23^. However, seldom studies reported which type of hypertension was associated with serum uric acid level. Daniel evaluated 125 children aged 6 to 18 years and concluded that uric acid levels were directly positive correlated with systolic and diastolic blood pressure^14^. Similarly, our results also found an association between hyperuricemia and higher diastolic blood pressure. Some studies stated the mechanism for the elevated serum uric acid level in children with primary hypertension. For example, hyperuricemia may affect arterial pressure by activating the renin-angiotensin system and reducing nitric oxide synthase activity^23^. Furthermore, a recent review including many clinical and experimental studies, summarized the association between serum uric acid concentration and a variety of cardiovascular and renal diseases, particularly in hypertension^24^. This review clearly demonstrated that an increasing serum uric acid level may be a useful biomarker of hypertension.

In our study, we also found hyperuricemia was associated with higher serum triglyceride concentrations, which was similar to previous studies^25, 26^. Those studies reported that hypertriglyceridemia was related to hyperuricemia independent of obesity and central body fat distribution. It is speculated that the synthesis of triglycerides needs NADPH, which results in increased uric acid production^27^. The biological mechanism of the relationship between serum triglyceride and uric acid is still unclear, and further studies *in vitro* and clinical are needed.

The major strength of our study is to report the prevalence of hyperuricemia in children aged from 3 to 6 years. To our knowledge, we are the first to examine the relationship between hyperuricemia and its risk factors, including BMI, SBP and DBP, the level of glucose, cholesterol and triglyceride in a preschool children population. Our study has certain limitations. First, we used the data from a cross-sectional survey to assess the risk indicators of hyperuricemia. Second, we cannot interpret well the causality between these risk indicators and hyperuricemia. In addition, the sample size of children aged less than 5 years was relatively small, which may influence the prevalence of hyperuricemia among little children. Further investigation on hyperuricemia and children aged less than 5 years is necessary. Third, the presence of hyperuricemia in parents was a better influencing factor than their history of gout for children hyperuricemia risk. However, in China, it is not universal for people to know clearly that they have hyperuricemia, but gout, as a kind of clinical symptoms of the disease, is easier to be known than hyperuricemia. So we investigated the history of gout in parents but not the presence of hyperuricemia in our study.

In conclusion, our findings of children aged from 3 to 6 years indicated that the prevalence of hyperuricemia among preschool age children in China was relatively high, especially in obese children. Several metabolic syndrome components were associated with the risk of hyperuricemia. Future studies in different population should be conducted to clarify the association between hyperuricemia and metabolic syndrome components among preschool age children.

## Methods

### Study population and settings

The city of Tianjin, located in Northern China, is the fourth largest city with a population of 14.1 million^28^ and is directly under the administration of the central government of China. Tianjin consists of 16 county-level administrative areas, including six central urban districts, one new urban district, four suburban districts and five rural districts. A cross-sectional survey was carried out in six central urban districts of Tianjin from March to June 2015. A cluster sampling was employed to obtain a random sample of preschool children. Three to six kindergarten schools were randomly selected from each central urban district and finally about 10% of kindergartens were included (26 kindergartens of 263). In Tianjin, children in kindergarten schools aged from 3 to 6 years old. All children (n = 7794) in selected kindergartens were invited to take part in our survey. Finally, a total of 4457 children, including 3391 (95.6% of 3547) children aged ≥ 5 years and 1066 (25.1% of 4247) children aged 3− < 5years, agreed to participate.

We sequentially excluded 24 children who did not have serum uric acid test and 360 children with missing variables required for this analysis. Finally, 4073 children were included in the analysis. The study protocols were guided by the Ethical Principles and Guidelines for the Protection of Human Subjects of Research and the study was approved by the Human Subjects Committee of the Tianjin Women’s and Children’s Health Center. An informed consent was obtained from all participants. The methods were carried out in accordance with the Declaration of Helsinki.

### Measurements

A questionnaire was completed by children’s parents at home. The questionnaire included information on the child’s birth date, sex, history of illness status; current health status, and parents’ socioeconomic factors (such as occupation, education, and family income), and parents’ history of gout and current health status (such as diabetes, dyslipidemia and hyperuricemia). Children who had tuberculosis, hepatitis, chronic bronchial catarrh, asthma of the lungs, or nephritis diagnosed by a doctor were classified as having a history of illness status. Current disease status was classified as yes for participants who reported having pneumonia, cold or fever during the past 30 days.

Weight, height and BP were measured by specially-trained pediatricians using the standardized protocol at the kindergarten clinic unit. Weight was measured to the nearest 0.01 kg using a digital scale (TCS-60, Tianjin Weighing Apparatus, China). Standing height was measured to the nearest 0.1 cm using a stadiometer (SZG-180, Shanghai Zhengdahengqi, China). Body mass index (BMI) was calculated by dividing weight in kilograms (kg) by the square of height in meters (m). Z-scores for weight, height, and BMI were calculated based on the World Health Organization (WHO) child growth standards (2–5 years old) and WHO child growth reference (5–19 years old)^29^. In order to make the data most useful for comparison, and to contribute to the understanding of international standard definitions of overweight and obesity, we used BMI to classify each child as “normal,” “overweight,” or “obesity”. Normal weight was defined as a BMI less than the 85th percentiles for age and gender using the WHO growth reference (<1035 Z score), overweight and obesity was defined as a BMI above the 85th percentile (≥1.035 Z score), and obesity was defined as a BMI above the 95th percentile (≥1.645 Z score)^29^. Sitting BP was measured from the right arm using automated sphygmomanometers (OMRON HBP-1300)^30^ after at least 5 minutes’ rest. The fifth Korotkoff sound was adopted for diastolic BP recording. Mean BP was calculated from 3 readings. Hypertension was defined based on the criteria recommended for Chinese children, i.e., systolic or diastolic BP ≥ 95th percentile for age and sex^31^.

All children provided a 0.5 mL peripheral blood sample from the ring finger after at least 8 hours of fasting at the kindergarten clinic unit^32^. The blood samples were immediately centrifuged and processed with an automatic device. Serum levels of total cholesterol, triglycerides, glucose and uric acid were measured enzymatically using an automatic analyzer (RX Daytona; Randox Laboratories Ltd, Antrim, Ireland)^33^. Due to the lack of practical guideline for hyperuricemia in preschool children, hyperuricemia was defined as 310 μmol/L (5.2 mg/dL, 90^th^ percentile in our sample) or higher. Hyperglycemia was defined as a fasting glucose level of 5.6 mmol/L or higher^34^. High total cholesterol and high triglycerides was defined as 5.18 mmol/L or higher and 1.70 mmol/L or higher, respectively^35^.

### Statistical analysis

As for the statistical analysis, quantitative variables were expressed as mean ± standard deviation and the quantitative variables as frequencies. Since the sample corresponds to a normal distribution, the One-way analysis of variance (ANOVA) was used to detect the difference among groups. To compare the difference of general characteristics between hyperuricemia and normal-level children, Chi-square test was used for categorical variables. Binary logistic regression was used to assess the associations between hyperuricemia and the related risk factors in univariable and multivariable analyses. The related risk factors included children’s age, sex, BMI, BP, serum levels of total cholesterol, triglycerides and glucose, gout history of parents, education of parents, family income and health history of parents. All statistical analyses were performed with PASW for Windows, version 20.0 (Statistics 20, SPSS, IBM, Armonk, NY). A p value of less than 0.05 for a two-tailed test was considered to be statistically significant.
